# FILE OF LIFE: OLDER ADULT PERSPECTIVES AND SUGGESTIONS TO INCREASE FOL USAGE

**DOI:** 10.1093/geroni/igac059.1808

**Published:** 2022-12-20

**Authors:** Kaye Dandrea, Teresa Williams, Marilyn Gugliucci

**Affiliations:** University of New England College of Osteopathic Medicine, Biddeford, Maine, United States; University of New England College of Osteopathic Medicine, Biddeford, Maine, United States; University of New England College of Osteopathic Medicine, Biddeford, Maine, United States

## Abstract

IntroductionIn a home emergency, an accurate patient medical history is vital for proper medical decision-making. One method to provide medical information in an emergency is through the FILE OF LIFE™ (FOL); a document distributed by local medical centers and public service organizations completed by community adults and placed on their refrigerator in a magnetic folder. To date, there was no known study, until this one, conducted with older adults to check the FOL design suitability for older adults; the goal of this project. MethodsOlder adults were recruited through the UNE COM Geriatrics Education Mentor (GEM) program for this pilot qualitative research. Twenty-one GEMs volunteered to participate in five 90-minute focus groups. Questions were specific for each section - Patient Identifiers, Emergency Contacts, Medical Data, Medical Conditions, Allergies, and Medical Insurance Information, and the usability of the FOL. Qualitative content analysis followed by NVivo 12+ analysis applied inter-rater reliability methods and changes for each FOL section were based on reaching data saturation. ResultsFILE OF LIFE™ comments (N=328) were coded to the following themes: Layout, Font Size, Spacing, and Section Ordering. Sections were edited accordingly with “Identifiers,” Medical Data, Medical Conditions, Allergies, and Emergency Contacts requiring substantive changes. Other minor changes were made throughout the form. ConclusionFurther research will ensure efficient usability by older adults and emergency response personnel. The FILE OF LIFE™, although a trusted resource for older community dwelling residents, has a chance to increase usability by incorporating the suggested changes from this research.

